# Predictors of Citation Rates in High-Impact Glioblastoma Clinical Trials

**DOI:** 10.7759/cureus.19229

**Published:** 2021-11-03

**Authors:** Ammer M Jamjoom, Abdulhadi Y Gahtani, Abdulhakim B Jamjoom

**Affiliations:** 1 Neurological Surgery, Leeds General Infirmary, Leeds, GBR; 2 Neurological Surgery, King Saud Bin Abdulaziz University for Health Sciences College of Medicine, Jeddah, SAU

**Keywords:** gbm, astrocytoma, grade iv glioma, glioblastoma, bibliometric analysis, high impact, citation prediction, citation rate, clinical trials, glioblastoma multiforme

## Abstract

Clinical trials are at the top of research study designs and tend to attract high citation numbers. Glioblastoma multiforme (GBM) is a multidisciplinary disease that continues to be the subject of peak research interest. In general, the literature relating to the predictors of citation rates in clinical trials remains limited. This review aims to identify the factors that influence citation numbers in high-impact GBM clinical trials. The 100 most cited GBM trials of any phase published from 1975 to 2019 were selected and reviewed. The primary analysis correlated citation numbers of articles with various trial and publication-related predictors using the Pearson correlation coefficient. The secondary analysis compared the mean citation numbers for different subgroups using the mean difference test. The median (range) citation number for the selected 100 trials was 349 (135-16,384). The primary analysis showed a significant correlation between citation numbers of articles and the study population (P = 0.024), trial phase (I-III) (P = 0.0427), and the impact factor (IF) of the journal (P < 0.0001). The secondary analysis demonstrated significantly higher mean citation numbers in all trials with the following features: study population ≥115 (P = 0.0208), phase III (P = 0.0372), treatment protocol including radiotherapy (P = 0.0189), temozolomide (TMZ) therapy (P = 0.0343), IF of the journal ≥14.9 (P = 0.02), and general medical journals (P = 0.28). We conclude that the most significant predictors of citation rates in high-impact GBM trials were the study population, trial phase, and journal’s IF. The treatment protocol was a positive predictor when it included the currently widely accepted treatment modalities (radiotherapy and TZM). Randomization, age of publication, as well as the numbers of arms, authors, centers, countries, and references were not significant predictors. Increasing awareness of the factors that could affect citations may help researchers undertaking clinical trials to enhance the academic impact of their work.

## Introduction and background

Citation-based metrics are used for calculating the impact factor (IF) of journals and for evaluating the academic productivity of researchers. The number of citations an article receives, also referred to as the citation rate, is arguably the most important measure of a study’s impact and clinical weight [1]. An analysis of the various article, journal, and author-related factors that may affect citation rates was reported in two publications [2,3]. These factors were also examined by other studies that focused on identifying the predictors of citations in published research relating to several specialties, including spine [4], neurosurgery [1], radiology [5], psychology [6], plastic surgery [7], cardiovascular [8], urology [9], and orthopedic surgery [10].

Randomized controlled trials (RCTs) are recognized as the pinnacle of clinical study designs and evidence-based medicine [11]. They are frequently published in high-impact journals and receive considerable visibility [11]. They are also likely to influence the opinions of clinicians, patients, and policymakers [11]. The association between study designs (RCTs and meta-analyses, in particular) and high citation numbers has been well documented in the literature [2,8-10]. However, clinical trials are not always randomized and vary in characteristics, completion, and publication rates [12]. Furthermore, studies analyzing citation patterns of clinical trials remain limited in the literature [11,13-15].

Glioblastoma multiforme (GBM) is a malignant primary central nervous tumor that represents an enigma to clinicians because of its aggressive and heterogeneous nature [16]. It is primarily a topic of oncology but includes the disciplines of neurosurgery, neurology, radiotherapy, basic science, and general medicine [16]. A recent bibliometric evaluation of high-impact GBM research did not address citation rates [16]. The objective of this review is to determine the different trial and publication-related predictors of citations in high-impact GBM clinical trials.

## Review

Methodology

PubMed and Google Scholar databases were searched in March 2021 for all GBM-related trials available in the literature. The inclusion criteria included highly cited clinical trials at any phase published from 1975 to 2019. We also searched the websites of the following journals: the New England Journal of Medicine, Lancet, Journal of American Medical Association (JAMA), Journal of Clinical Oncology, Neuro-oncology, and Journal of Neurosurgery. The main keywords for the literature search were “Glioblastoma,” “GBM,” “Glioblastoma Multiforme,” “Grade IV Glioma,” “Trials,” and “Randomized Controlled Trials.” Articles were assessed for suitability using the abstract, and the full text was reviewed in case of ambiguity. Using article citation numbers provided by Google Scholar, the 100 most cited GBM trials were identified. Trials that reported extended findings from an earlier trial were included if they received high citation numbers. To minimize bias, two authors conducted independent searches and prepared separate lists of the most cited articles. The two lists were compared, and any discrepancies were resolved by consensus. In view of the regular changes in citation numbers, the search findings on a single day (April 01, 2021) were documented and used for analysis. In addition, journal IFs were obtained from the journals’ websites for 2019 as these were the latest available at the time of the analysis. The selected trials were analyzed, and the information relating to the characteristics of the trials and publications was collected. The data were used to generate descriptive statistics relating to the 100 high-impact GBM clinical trials.

The primary analysis correlated the total citation numbers for the various studies with the following trial and publication-related predictors: study population; randomization; the number of arms; phase; GBM status; treatment modality used in any of the trial arms [chemotherapy (including temozolomide (TZM), nitrosourea, bevacizumab (BVZ), others); radiotherapy (including electrotherapy and proton/neutron irradiation); surgery; local treatment (chemotherapy, immunotherapy, hyperthermia) and immunotherapy]; trial duration in months; duration from publication in years; publishing journal’s IF and field (oncology, general medicine, neurosurgery); and the number of authors, centers, countries, and references listed on the publication. The correlation analysis was done by calculating the Pearson correlation coefficient (R) using Social Sciences Statistics [17], and significance was determined at a P-value of ≤0.05.

For further evaluation of the impact of the chosen predictors, a secondary analysis was conducted by calculating and comparing the mean citation numbers [±standard deviation (SD)] between different subgroups. The median was taken as a cut-off point in the numerical parameters, with the following comparisons: study population [<115 vs. ≥115]; randomization (yes vs. no); arms (1 vs. 2-4); phase (I, I-II, II, II-III vs. III); GBM status (newly diagnosed vs. recurrent); treatment modality [chemotherapy vs. all others, TZM vs. all others, nitrosourea vs. all others, BVZ vs. all others, radiotherapy vs. all others, surgery and local treatment vs. all others, and immunotherapy vs. all others]; study duration in months (<30 vs. ≥30); duration from publication in years (<13 vs. ≥13); journal’s IF (<14.9 vs. ≥14.9); journal’s field (general medicine vs. others, oncology vs. others); the number of authors (<14 vs. ≥14); the number of centers (<10 vs. ≥10); the number of countries (1 vs. >1); and the number of references (<30 vs. ≥30). The statistical analysis was carried out by calculating the mean difference (MD) using MedCalc [18], and significance was determined at a P-value of ≤0.05.

Results

The median (range) and mean (±SD) total citation numbers for the 100 most cited GBM trials were 340 (135-16,284) and 825 (±1,828), respectively. An analysis of the trials is shown in Appendices. The median (range) and findings relating to the various trial and publication parameters are summarized in Tables 1, 2. The 100 trials were published in the following journals: Journal of Clinical Oncology, 22%; Lancet Oncology and Lancet, 12%; Neuro-Oncology, 10%; New England Journal of Medicine, 8%; Journal of Neurosurgery, 8%; International Journal of Radiation Oncology Biology Physics, 5%; JAMA and JAMA Oncology, 3%; Nature and Nature Medicine, 3%; British Journal of Cancer, 3%; Clinical Cancer Research, 3%; and miscellaneous, 23%. The mean IF for oncological, general medical, and neurosurgical journals were 20.1, 40.5, and 4, respectively. Of the selected 100 trials, the treatment protocols included chemotherapy using one or more agents in 73% (TZM: 31%, nitrosourea: 17%, BVZ: 12%, erlotinib and gefitinib: 7%, irinotecan: 5%, and others: 30%), radiotherapy in 38% (including tumor treatment fields: 2%, photodynamic therapy: 2%, accelerated proton/photon irradiation: 1%, and neutron capture therapy: 1%), surgery and local treatment in 13%, and immunotherapy in 9%. Tables 1, 2 summarize the primary analysis correlation results between citation numbers and the various predictors. A significant correlation was observed between citation numbers and study population (R = 0.226; P = 0.024), journal’s IF (R = 0.4085; P < 0.0001), and trial phase (R = 0.2031; P = 0.0427). No significant correlation was found between citation numbers and randomization, the number of trial arms, GBM status, treatment protocols, trial duration, duration from publication, and the number of authors, centers, countries, and references.

**Table 1:** Summary of the median (range) results for several predictors as well as their correlation analysis with citation numbers. *P-values ≤0.05 are significant. IF: impact factor

Parameter	Median (range)	R-value	P-value
Study population	115 (8–1578)	0.226	0.024*
Trial duration (months) [N = 82]	30 (7–113)	−0.0559	0.6179
Duration from publication (years)	13 (2–43)	0.0688	0.4964
Journal’s IF	14.9 (1.6–74.4)	0.4085	<0.0001*
Number of authors	14 (3–69)	0.0135	0.8939
Number of centers	10 (1–58)	0.0782	0.4393
Number of countries	1 (1–14)	0.1901	0.0582
Number of references	30 (9–60)	0.0238	0.8142

**Table 2:** Summary of the findings for several predictors as well as their correlation analysis with citation numbers. *P-values ≤0.05 are significant. GBM: glioblastoma multiforme

Parameter	Variables	Finding (%)	R-value	P-value
Randomization	Yes	59%	0.1836	0.0675
	No	41%		
Number of arms	1	37%	0.0847	0.402
	2	47%		
	3	7%		
	4	9%		
Phase	I	8%	0.2031	0.0427*
	I-II	8%		
	II	36%		
	II-III	2%		
	III	46%		
GBM status	New	61%	0.1242	0.2183
	Recurrent	39%		
Treatment protocols	Chemotherapy	73%	0.1782	0.0761
	Radiotherapy	38%		
	Surgery and/or local treatment	13%		
	Immunotherapy	10%		
Journal’s field	General medical	25%	0.0989	0.3276
	Oncological	67%		
	Neurosurgical	8%		

Table 3 summarizes the results of the mean difference comparative secondary analysis between the various subgroups. The value of the SD was greater than the mean in most reported findings in Table 3, indicating that the data had skewed distribution due to the wide range of variation in citation numbers among the selected articles. A significantly higher mean citation number was observed for trials with a study population ≥115 compared to <115 (1,248 vs. 404; P = 0.0208), trials that reported phase III results compared to others (1,223 vs. 460; P = 0.0372), trials in which the treatment protocol included radiotherapy compared to others (1,423 vs. 519; P = 0.0189), trials in which the treatment protocol included TZM compared to others (1,414 vs. 573; P = 0.0343), trials that were published in journals with IF ≥14.9 compared to <14.9 (1,251 vs. 402; P = 0.02), trials that were published in high-impact general medicine journals compared to others (1,521 vs. 594; P = 0.28). No significant difference was found in the mean citation numbers between the two subgroups relating to randomization, the number of trial arms, GBM status, treatment protocols that included chemotherapy in general, nitrosourea, BVZ, surgery, local treatment, and immunotherapy, as well as trial duration, the period from publication, and the number of authors, centers, countries, and references.

**Table 3:** Summary of the comparative analysis of the citation numbers for the various predictor subgroups. *P-values ≤0.05 are significant.

Feature	Variables	Number	Mean citation numbers (±SD)	Mean difference	P-value
Study population	<115	50	404 (±349)	844	0.0208*
	≥115	50	1248 (±2,515)		
Randomization	Yes	59	1,094 (±2,319)	−670	0.0703
	No	41	424 (±384)		
Number of arms	1	37	420 (±374)	666	0.0768
	2, 3, 4	63	1,086 (±2,302)		
Phase	I, I-II, II, II-III	54	460 (±454)	763	0.0372*
	III	46	1,223 (±2,563)		
GBM status	New	61	1,008 (±2,305)	-465	0.2186
	Recurrence	39	543 (±512)		
Treatment protocols	Chemotherapy	73	519 (±590)	420	0.1219
	All others	27	939 (±2,102)		
	Temozolomide	31	1,414 (±3,170)	−841	0.0343*
	All others	69	573 (±595)		
	Nitrosourea	17	578 (±589)	299	0.05435
	All others	83	877 (±1,997)		
	Bevacizumab	12	1,016 (±822)	−216	0.7043
	All others	88	800 (±1,935)		
	Radiotherapy	38	1,423 (±2,990)	−904	0.0189*
	All others	62	519 (±558)		
	Surgery and/or local treatment	13	722 (±790)	70	0.8976
	All others	87	842 (±1,947)		
	Immunotherapy	10	266 (±89)	621	0.3126
	All others	90	887 (±1,926)		
Trial duration (months) [N = 82]	<30	40	1,194 (±2,796)	−569	0.2004
	≥30	42	625 (±574)		
Period from publication (years)	<13	48	584 (±923)	457	0.2156
	≥13	52	1,041 (±2,380)		
Journal’s IF	<14.9	50	402 (±396)	849	0.02*
	≥14.9	50	1,251 (±2,507)		
Journal’s field	General medicine	25	1,521 (±3,346)	−927	0.028*
	All others	75	594 (±813)		
	Oncology	67	611 (±835)	652	0.0952
	All others	33	1,263 (±2,950)		
Number of authors	<14	45	536 (±590)	528	0.0968
	≥14	55	1,064 (±2042)		
Number of centers	<10	50	492 (5±09)	668	0.0687
	≥10	50	1,160 (±2,515)		
Number of countries	1	56	566 (±608)	592	0.11
	>1	44	1,158 (±2,663)		
Number of references	<30	49	743 (±1,030)	163	0.6595
	≥30	51	906 (±2,376)		

Discussion

GBM has been the focus of substantial clinical and scientific research aimed at discovering a treatment modality that can significantly improve survival [13]. A recently reported analysis of the 100 most cited GBM publications included 27 clinical studies, 19 of which were trials that were included in this study (Appendices Table 4) [1]. A review of the 44 neurosurgical RCTs in high-impact journals that were published did not contain any GBM trials [11]. None of these publications assessed citation patterns.

The mean citation number for the 100 most cited GBM trials was 825, which was higher than the mean citation of 198 for the 100 most cited meningioma articles [19]. However, it is slightly lower than the reported median citation number of 935 for the 100 most cited GBM articles [16]. This finding is not surprising as the mentioned review covered a bigger pool of GBM studies that included 52 basic science articles [16]. The latter are recognized to be associated with high citation numbers [2,10,16]. Variation in citation rates according to study topic or subject is well recognized in the literature relating to neurosurgery [1], spine [4], plastic surgery [7], and urology [9]. It is generally accepted that disciplines differ in their citation practices and that certain topics or subject areas may be cited more than others [2]. Moreover, the number of citations is influenced by the size of the literature in the field [2].

In this analysis, a significant association with the study population was observed in both primary and secondary analysis, implying that the study population was a firm predictor of citation rates in GBM clinical trials. Similar findings relating to the study population were reported by other studies [2,4,6,9,20]. A significant correlation with the trial phase was also seen in both primary and secondary analysis, indicating that being a phase III trial was a solid predictor of citation rates. However, citation rates were not affected by randomization which is surprising as the correlation between RCT-type studies and bigger citation numbers is well reported in the literature [1,8-10]. This finding could be unique to the field of GBM research or could be related to the relatively limited number of articles selected in this review. Citation rates were not affected by the number of arms, status of GBM, and duration of the trial. The lack of impact of certain features of study designs on citation numbers was also reported by others [2,20].

In this study, the primary analysis did not reveal a correlation between treatment protocols and citation rates. However, the secondary analysis demonstrated significantly higher citation rates in trials in which the treatment protocol included radiotherapy and TZM. This probably reflects the current widely accepted standard treatment for newly diagnosed GBM, which includes surgery followed by concurrent radiotherapy with TZM and further adjuvant TZM [21,22]. No significant association was observed between citation rates and treatment protocols that included chemotherapy in general. This probably relates to the wide-ranging chemotherapeutic agents used in the studies and their mixed efficacy. Furthermore, chemotherapy in the general group included older studies that were conducted before the use of standard TZM in the first-line setting. The lack of association between citation numbers and treatment protocols including BVZ, nitrosourea, surgery and local treatment, and immunotherapy may be influenced by the limited number of trials that focused on the treatment modality. However, it could reflect their undetermined role in the management of GBM [21,22]. Citation rates were also not affected by the duration from the time of publication (age of the study). This is not unusual as the study covered a long period (43 years). It is recognized that the number of citations increases in the first year after publication to reach a peak and then they are less cited as time passes [2]. The latter could be because the article’s information becomes outdated with time [2].

In this article, significant association with journal’s IF was observed in both primary and secondary analyses showing that journal’s IF was a strong predictor of citation rates in GBM clinical trials. Similar findings relating to the journal’s IF were reported by other studies [1,2,15]. Furthermore, the secondary analysis demonstrated significantly higher citation rates in trials that were published in general medical journals. This was expected as the group of general medical journals in this study had a much higher mean IF than the oncological and neurosurgical groups (40.5 vs. 20.1 and 4, respectively). The association between journals and higher citations rates was documented in the literature in association with spine [4], plastic surgery [7], and transplantology [23].

In this review, no significant link was found between citation rates of GBM clinical trials and the numbers of authors, centers, countries, and references. A similar finding was reported by other studies [2,20]. However, in the literature, several publications have identified the number of authors as a significant predictor of citation [1,7,15]. Significant relationships have also been reported between the international and national collaboration of authors, the number of organizations, the number of countries producing the paper, and the frequency of citations [1,2]. A positive relationship with the number of references was reported by some studies [20]. Furthermore, some studies suggested that a proportion of variance in the number of citations an article receives can be explained by seemingly superficial factors unrelated to the content of the article such as the title, the number of authors, the number of references, the number of sentences in the abstract, the presence of a colon in the title, and the number of pages [24,25].

The countries where the GBM clinical trials originated were not examined in this study. It has been reported that the country of origin can be a positive predictor of citation counts in research relating to spine [4], radiology [5], and urology [9]. Furthermore, a recent publication [3] investigated the impact of 66 factors on citations using samples of articles from 18 leading Chinese library and information science journals. They found 46 factors were significantly associated with citations. They also observed the most significant factors to be the number of downloads, the number of citations in the first five years, the author being an independent researcher, and the percentage of monographs in the references. Several other potential predictors were not addressed in this study that were examined by other studies. These include increasing visibility through open access [5], selection for press release [26], funding [14,20], disclosure of conflict of interest [7], statistically significant results [20], and the trial being referenced in ClinicalTrials.gov [27].

Limitations

There are several limitations to this study. The study relied on the precision of online search engines PubMed and Google Scholar. The selection of the 100 trials was based on their total citations at a certain point which was likely to change relatively quickly. This could have influenced the inclusion or exclusion of a few of the lower-impact trials. The wide duration from publication may have affected the citations of older trials. There may have been potential errors in the subgrouping of the treatment protocols. Moreover, variation in author affiliation may have affected the number of centers. Collaborators were not counted in the number of authors, centers, and countries. In addition, the impact of self-citation on citation numbers was not examined.

## Conclusions

Clinical trials are the pinnacle of research study designs and tend to attract high citation numbers. GBM is a multidisciplinary disease that continues to be a subject of peak research interest. The literature relating to the predictors of citation rates in clinical trials remains limited. The most consistent predictors of citation rates in GBM clinical trials were study population, trial phase, and journal IF. The treatment protocol was a positive predictor when it included the currently widely accepted treatment modalities (radiotherapy and TZM). Randomization, age of publication, as well as the numbers of arms, authors, centers, countries, and references were not significant predictors. Increasing awareness of the factors that could affect citations may help researchers undertaking clinical trials to enhance the academic impact of their work. Further research on the predictors of citations in trials related to other pathological entities is encouraged.

## Appendices

**Table 4:** Analysis of the 100 high-impact GBM clinical trials. GBM: glioblastoma multiforme; Phas: phase; Rand: randomization; IF: impact factor; Size: sample size; RT: radiotherapy; chemo: chemotherapy;  TZM: temozolomide; BVZ: bevacizumab; BCNU: carmustine; MeCCNU: semustine; HSV: herpes simplex virus; TTF: tumor treatment field; DTIC: dacarbazine; VCT: vincristine; NDV: Newcastle disease virus; PCZ: procarbazine; HU: hydroxyurea; VM-26: epipodophyllotoxin; ALA: amino-levulenic acid; PDT: photodynamic therapy; EIAED: enzyme inducing antiepileptic drugs; IV: intravenous; IA: intra-arterial; DFMO: difluromethylornithine; N Engl J Med: New England Journal of Medicine; J Clin Oncol: Journal of Clinical Oncology; J Neurosurg: Journal of Neurosurgery; J Natl Cancer Inst: Journal of the National Cancer Institute; Clin Cancer Res: Clinical Cancer Research; Br J Cancer: British Journal of Cancer; JAMA: Journal of American Medical Association; Eur J Cancer: European Journal of Cancer; Int J Radiat Oncol Biol: International Journal of Radiation Oncology-Biology-Physics; Ann Intern Med: Annals of Internal Medicine; Cancer Immunol: Cancer Immunology Immunotherapy; Lasers Med Sci: Laser in Medical Science; J Neurooncol: Journal of Neuro-oncology; Cancer Chemother: Cancer Chemotherapy and Pharmacology,  J Transl Med: Journal of Translational Medicine, Am J Clin Oncol: American Journal of Clinical Oncology; J Environ Pathol: Journal of Environmental Pathology and Toxicology and Oncology; Mol Cancer Ther: Molecular Cancer Therapeutics

No.	First author	Citation	Cites	IF	Size	Rand	Phas	Treatment protocols
1	Stupp	N Engl J Med 2005; 352: 987-96	16,284	74.7	573	Yes	3	RT vs. RT ± TZM
2	Hegi	N Engl J Med 2005; 352: 997-1003	6,376	74.7	206	Yes	3	RT vs. RT ± TZM
3	Stupp	Lancet Oncology 2009; 10: 459-66	5,856	33.8	573	Yes	3	RT vs. RT ± TZM
4	Stummer	Lancet Oncology 2006;7: 392-401	2,857	33.8	270	Yes	3	Surgical resection using fluorescence vs. white light
5	Friedman	J Clin Oncol 2009; 27: 4733-40	2,390	33	167	Yes	2	BVZ + irinotecan vs.BVZ
6	Gilbert	N Engl J Med 2014; 370: 699-708	2,020	74.7	621	Yes	3	BVZ vs. Placebo
7	Walker	J Neurosurg 1978; 49: 333-43	1,997	4	303	Yes	3	RT vs. BCNU vs. BCNU ± RT vs. supportive care
8	Chinot	N Engl J Med 2014; 370: 709-22	1,837	74.7	921	Yes	3	Placebo + RT + TZM vs. BVZ + RT + TZM
9	Walker	N Engl J Med 1980; 303: 1323-29	1,817	74.7	358	Yes	3	MeCCNU vs. RT vs. BCNU ± RT vs. MeCCNU ± RT
10	Kreisl	J Clin Oncol 2009; 27: 740-5	1,539	33	48	No	2	BVZ then BVZ + irinotecan (1 arm)
11	Vredenburgh	J Clin Oncol 2007; 25: 4722-9	1,528	33	35	No	2	BVZ + irinotecan (1 arm)
12	Brem	Lancet 1995; 345: 1008-12	1,502	60.4	222	Yes	3	Intraoperative carmustine vs. placebo polymers
13	Curran	J Natl Cancer Inst 1993; 85: 704-10	1,406	11.6	1578	Yes	2-3	RT (multiple) vs. RT + chemo (nitrosourea/others)
14	Westphal	Neuro-Oncology 2003; 5: 79-88	1,333	10.3	240	Yes	3	Intraoperative carmustine wafers vs. placebo
15	Vredenburgh	Clin Cancer Res 2007; 13: 1253-9	1,148	10.1	32	No	2	BVZ + irenotican (1 arm)
16	Yung	Br J Cancer 2000; 83: 588-93	1,093	5.8	225	Yes	2	TZM vs. procarbazine
17	Markert	Gene Therapy 2000; 7: 867-74	1,041	4.1	21	No	1	HSV G207 inoculation (1 arm)
18	Wong	J Clin Oncol 1999; 17: 2572-8	989	33	458	No	2	Multiple regimes (8 trials)
19	Malmström	Lancet Oncology 2012;13: 916-26	945	33.8	342	Yes	3	TZM vs. RT vs. hypofractionated RT
20	Stupp	J Clin Oncol 2002; 20: 1375-82	942	33	62	No	2	TZM + RT then TZM (1 arm)
21	Wick	Lancet Oncology 2012; 13: 707-15	911	33.8	412	Yes	3	TZM vs. RT
22	Hegi	Clin Cancer Res 2004; 10: 1871-4	847	10.1	38	No	2	TZM + RT then TZM (1 arm)
23	Rich	J Clin Oncol 2004; 22: 133-42	788	33	75	No	2	Gefitinib (1 arm)
24	Roa	J Clin Oncol 2004; 22: 1583-8	783	33	100	Yes	2	Standard RT vs. short course RT
25	Stupp	JAMA 2015; 314: 2535-43	774	45.5	315	Yes	3	TTF + TZM vs. TZM
26	Galanis	J Clin Oncol 2005; 23: 5294-304	772	33	65	No	2	Temsirolimus (1 arm)
27	Keime-Guibert	N Engl J Med 2007; 356: 1527-35	756	74.7	85	Yes	3	Focal RT vs. supportive care
28	Stupp	JAMA 2017; 318: 2306-16	696	45.5	695	Yes	3	TTF + TZM vs. TZM
29	Gilbert	J Clin Oncol 2013; 31: 4085-91	677	33	833	Yes	3	TZM vs. Dose-dense TZM
30	Chang	Cancer 1983; 52: 997-1007	670	5.8	554	Yes	3	RT vs. RT + Boost vs. RT + BCNU vs. RT + MeCCNU + DTIC
31	Stupp	Lancet Oncology 2014; 15: 1100-8	666	33.8	545	Yes	3	Cilengitide vs. placebo
32	Cloughesy	PLoS Medicine 2008; 5: e8	595	10.5	15	No	1	Rapamycin + salvage surgical resection (1 arm)
33	Taal	Lancet Oncology 2014; 15: 943-53	577	33.8	153	Yes	2	BVZ vs. lomustine vs. BVZ + lomustine
34	Stupp	Eur J Cancer 2012; 48: 2192-202	564	7.3	237	Yes	3	Novo TTF -100 A vs. active chemotherapy
35	van den Bent	J Clin Oncol 2009; 27: 1268-74	540	33	110	Yes	2	Erlotinib vs. TZM or carmustine
36	Mirimanoff	J Clin Oncol 2006; 24: 2563-9	528	33	573	Yes	3	RT vs. RT ± TZM
37	Gorlia	Lancet Oncology 2008; 9: 29-38	521	33.8	573	Yes	3	RT vs. RT ± TZM
38	Levin	Int J Radiat Oncol Biol 1990; 18: 321-4	500	5.9	133	Yes	3	RT + BCNU vs. RT + PCZ + CCNU + VCT
39	Kristiansen	Cancer 1981; 47: 649-52	488	5.8	118	Yes	3	Placebo vs. RT vs. RT + bleomycin
40	Perry	N Engl J Med 2017; 376: 1027-37	477	74.7	562	Yes	3	RT (short course) vs. RT (short course) ± TZM
41	Sneed	Int J Radiat Oncol Biol 1998; 40: 287-95	458	5.9	112	Yes	2-3	Brachytherapy + interstitial hyperthermia vs. brachytherapy
42	Sotelo	Ann Intern Med 2006; 144: 337-43.	452	21.3	30	Yes	3	Placebo vs. choroquine
43	Brada	Annals of Oncology 2001; 12: 259-66	438	18.3	138	No	2	TZM (1 arm)
44	Weller	Lancet Oncology 2017; 18: 1373-85	437	33.8	745	Yes	3	Rindopepimut vs. placebo
45	Batchelor	J Clin Oncol 2013; b31: b3212-8	408	33	325	Yes	3	Cediranib vs cediranib+ lomustine vs lomustine+ placebo
46	Keskin	Nature 2019; 565: 234-9.	397	42.8	8	No	1	Nanoantigen vaccine (1 arm)
47	Wick	N Engl J Med 2017 ; 377: 1954-63	382	74.7	437	Yes	3	Lomustine vs. lomustine + BVZ
48	Kunwar	Neuro-Oncology 2010; 12: 871-81	381	10.3	296	Yes	3	Intraoperative cintredekin besudotax vs. Glaidel wafers
49	Freeman	Molecular Therapy 2006; 13: 221-8	362	9	14	No	1-2	IV NDV-HUJ oncolytic virus (1 arm)
50	Cloughesy	Nature Medicine 2019; 25: 477-86	340	36.2	35	Yes	2	Pembrolizumab vs. adjuvant
51	Rosenfeld	Autophagy 2014;10: 1359-68	340	9.8	92	No	1-2	Hydroxychloroquine (1 arm)
52	Wisoff	J Neurosurg 1998; 89: 52-9	325	4	131	No	3	Biopsy vs. partial vs. subtotal vs. near-total vs. total excision
53	Galanis	J Clin Oncol 2009; 27: 2052-8	321	33	66	No	2	Vorinostat (1 arm)
54	Shapiro	J Neurosurg 1989; 71: 1-9	312	4	571	Yes	3	RT + BCNU vs RT + BCNU + PCZ vs RT + BCNU + HU + PCZ + VM-26
55	Taphoorn	Lancet Oncology 2005; 6: 937-44	309	33.8	573	Yes	3	RT vs. RT ± TZM
56	Phu- phanich	Cancer Immunol 2013; 62: 125-35	308	5.4	17	No	1	Multi-epitope-pulsed dendritic cell vaccine (1 arm)
57	Brown	J Clin Oncol 2008; 26: 5603-9	288	33	97	No	1-2	Erlotinib + TZM (1 arm)
58	Ahmed	JAMA Oncology 2017; 3: 1094-101	286	24.8	17	No	1	HER2- specific chimeric antigen receptor-modified virus-specific T cells (1 arm)
59	Weller	Neurology 2011; 77: 1156-64	280	8.1	573	Yes	3	RT vs. RT ± TZM
60	Hilf	Nature 2019; 565: 240-5	279	42.8	15	No	1	Actively personalized vaccination (1 arm)
61	Schuster	Neuro-Oncology 2015; 17: 854-61	273	10.3	65	Yes	2	Rindopepimut (CDX-110) (1 arm)
62	Raizer	Neuro-Oncology 2010; 12: 95-103	272	10.3	96	No	2	Erlotinib (1 arm)
63	Shapiro	J Neurosurg 1992;76:772-81	259	4	448	Yes	3	IV BCNU ± 5-Flourouracil vs. IA BCNU ± 5-Fl-uracil
64	Groves	J Clin Oncol 2002; 20: 1383-8	257	33	44	No	2	TZM and marimastat (1 arm)
65	Fitzek	J Neurosurg 1999; 91: 251-60	255	4	23	No	2	Accelerated proton/photon irradiation (1 arm)
66	Eljamel	Lasers Med Sci 2008;23: 361-7	255	2.6	27	Yes	3	ALA + photofrin *surgical resection+ PDT vs. control
67	Sandmann	J Clin Oncol 2015; 33: 2735-44	234	33	349	Yes	3	BVZ + TZM + RT vs. TZM + RT + placebo
68	Weller	Clin Cancer Res 2015; 21: 2057-64.	223	10.1	105	Yes	3	TZM vs. TZM (Dose intensified)
69	Hasselbalch	Neuro-Oncology 2010; 12: 508-16.	215	10.3	43	No	2	BVZ + irinotecan + cetuximab (1 arm)
70	Peereboom	J Neurooncol 2010; 98: 93-9	213	3.3	27	No	2	TZM + erlotinib + RT (1 arm)
71	Reardon	Clin Cancer Res 2006; 12: 860-8	207	8	34	No	1	Gefitinib + sirolimus + EIAED vs. Gefitinib + sirolimus
72	Izumoto	J Neurosurg 2008; 108: 963-71	190	4	21	No	2	Wilms tumor 1 peptide vaccination (1 arm)
73	Coderre	Neuro-oncology 1997; 33:141-52	189	3.3	18	No	1-2	Boron neutron capture therapy (1 arm)
74	Thiessen	Cancer Chemother 2010; 65: 353-61	188	3.1	17	No	1-2	Lapatinib (GW572016) (1 arm)
75	Liau	J Transl Med 2018; 16: 142	186	4.2	331	Yes	3	Autologous tumor lysate dendritic cell vaccine (DCVax-L) (1 arm)
76	Nelson	Int J Radiat Oncol Biol 1993; 25: 193-207	182	5.9	466	Yes	1-2	RT (three doses) + BCNU
77	Chinot	Advances in Therapy 2011; 28: 334-40	182	3.3	920	Yes	3	BVZ+ TZM + RT vs. TZM + RT + placebo
78	Iwamoto	Neuro-Oncology 2010; 12: 855-61	181	10.3	35	No	2	Pazopanib (1 arm)
79	Sloan	J Neurosurg 2013; 118: 1202-19	177	4	10	No	1	NeuroBlate *local thermotherapy (1 arm)
80	Prados	Neuro-Oncology 2003; 5: 96-103	174	10.3	122	Yes	2	RMP-7 + carboplatin vs. placebo+ carboplatin
81	Clarke	J Clin Oncol 2009; 27: 3861-7	174	33	85	Yes	2	Dose dense TZM vs. metronomic TZM
82	Larner	Am J Clin Oncol 1998; 21: 579-83	172	3.1	18	No	1-2	Lavostatin + RT vs. lavostatin (1 arm)
83	Grana	Br J Cancer 2002; 86: 207-12	167	5.8	37	No	1-2	Yttrium-90-biotin vs. no adjuvant
84	Friday	Neuro-Oncology 2012; 14: 215-21	166	10.3	37	No	2	Vorinostat + bortezomib (1 arm)
85	Westphal	Lancet Oncology 2013; 14: 823-33	164	33.8	250	Yes	3	Perilesional sitimagene ceradenovec + IV ganciclovir vs. standard care
86	Herrlinger	J Clin Oncol 2006; 24: 4412-7	162	33	31	No	2	Lomustine + TZM + RT (1 arm)
87	Gállego Pérez Larraya	J Clin Oncol 2011; 29: 3050-5	161	33	70	No	2	TZM (1 arm)
88	Grossman	J Clin Oncol 2003; 21: 1485-91	157	33	223	Yes	3	Carmustine + cisplatin+ RT vs. carmustine + RT
89	Stepp	J Environ Pathol 2007; 26: 157-64	157	1.6	19	Yes	3	ALA + interstitial PDT vs. white light
90	Nabors	Neuro-Oncology 2015; 17: 708-17	157	10.3	265	Yes	2	Cilengitide vs. intensive cilengitide vs. standard
91	Prados	Int J Radiat Oncol Biol 2001; 49: 71-7	155	5.9	231	Yes	3	Hyperfractionated RT ± DFNO vs. standard RT ± DFMO
92	Hegi	Mol Cancer Ther 2011; 10: 1102-12	155	5.6	22	No	2	Gefitinib vs control
93	Herrlinger	Lancet 2019; 393: 678-88	154	60.4	129	Yes	3	Lomustine + TZM vs. TZM
94	Weller	J Clin Oncol 2003; 21: 3276-84	153	33	375	Yes	3	Nimustine + teniposide vs nimustine + Cytarabine
95	Brandes	Neurology 2004; 63: 1281-4	146	8.1	40	No	2	BCNU (1 arm)
96	Bloom	Br J Cancer 1973; 27: 253-67	142	5.8	62	Yes	2	irradiated autologous tumor cells vs. placebo
97	Narayana	J Neurosurg 2012; 116: 341-5	137	4	51	No	2	BVZ + TZM (1 arm)
98	Prados	Int Radiat Oncol Biol 2004; 58: 1147-52	137	5.9	134	Yes	3	RT + PCV + BUdR vs. RT + PCV
99	Suchorska	Neuro-Oncology 2016; 18: 549-56	137	10.3	71	No	2	Redo surgery for recurrence vs. no surgery
100	Grossman	J Clin Oncol 2009; 27: 4155-61	135	33	72	No	2	Talampanel + TZM + RT (1 arm)
